# Microfiltration of Post-Fermentation Broths: Long-Term Studies on the Use of Modules with Polymeric Membranes

**DOI:** 10.3390/membranes15110345

**Published:** 2025-11-19

**Authors:** Wirginia Tomczak, Marek Gryta

**Affiliations:** 1 Faculty of Chemical Technology and Engineering, Bydgoszcz University of Science and Technology, 3 Seminaryjna Street, 85-326 Bydgoszcz, Poland; tomczak.wirginia@gmail.com; 2 Faculty of Chemical Technology and Engineering, West Pomeranian University of Technology in Szczecin, Piastów Ave. 17, 70-310 Szczecin, Poland

**Keywords:** fermentation, fouling, glycerol, membrane cleaning, microfiltration, capillary module, spiral wound module

## Abstract

A primary target in the long-term microfiltration (MF) of fermentation broths is to ensure the high-quality permeate and stable system operation. This can be achieved by the choice of the most profitable membrane material and development of an effective membrane cleaning procedure. However, selecting the appropriate module configuration is also of key importance. This study assessed the suitability of capillary and spiral-wound modules for MF 1,3-propanediol (1,3-PD) fermentation broths, which were clarified only by 2 h of sedimentation. The obtained results demonstrated that the MF process allowed the removal of almost 100% of suspended solids from a feed. Consequently, the obtained high-quality permeate was characterized by the turbidity of 0.4–0.7 NTU. Fouling was mitigated by membranes’ washing with NaOH solution; hence, chemically resistant polytetrafluoroethylene (PTFE) and polypropylene (PP) membranes were installed in the modules. In order to determine dominant fouling mechanism, the Hermia model was applied. It has been shown that a decrease in the process performance was mainly caused by the formation of a cake layer on the membrane’s surface. A significant amount of the deposit also formed inside the mesh filling of the module channel, which excluded the use of spirally wound modules for the MF broth pretreated only by sedimentation. To avoid this phenomenon, the capillary PP membranes (diameter 1.8 mm) were applied. During long-term tests (over 700 h) membranes were periodically cleaned with the 1% NaOH solution, which removed most of the foulants. However, in this case, residual deposits formed by silicates remained on the membrane surface, requiring an additional membrane cleaning method. Finally, it has been noted that the PP membranes showed an excellent resistance to the frequent exposure to the foulants present in the fermentation broths and the alkaline agent.

## 1. Introduction

Recently, there has been upsurge of interest in the bioconversion of biomass into value-added products with a high commercial application such as 1,3-propanediol (1,3-PD). However, obtaining a main product characterized by high purity is a great challenge. This is mainly due to the fact that fermentation broths are highly complex, medium containing, apart from the main product, bacterial cells, byproducts, and proteins, as well as inorganics and multivalent ionic compounds [1,2]. For this reason, in downstream processing (DSP), several steps are required, including, for instance, microfiltration (MF), ultrafiltration (UF), nanofiltration (NF), and reverse osmosis (RO).

Obviously, as an initial separation step, microorganisms and suspensions present in a fermentation broth need to be removed. For this purpose, MF can be applied. It is important to note that due to the high turbidity of the feed, broth pretreatment is often required [3]. However, this increases the separation costs; hence, the possibility of MF broth being pretreated only by sedimentation was investigated in this study.

In recent years, MF of various fermentation broths has been extensively researched. Indeed, the use of membranes made of many different materials, for instance polypropylene (PP) [4,5,6], polyvinylidene fluoride (PVDF) [3,7], polysulfone [8], cellulose acetate–polysulfone blend [9], and ceramic [5,10,11,12,13,14], for clarifying fermentation broths has been reported in the literature. However, to the best of our knowledge, the literature has focused mainly on short-term processes lasting up to several hours. This can be explained by the fact that the most important limitation to the implementation of the long-term MF process lies in membrane fouling. Most of the aforementioned studies used polymeric membranes mounted in dead-end laboratory-scale installations. Therefore, the process flow was not evaluated using industrial designs such as capillary or spiral-wound modules. Therefore, in the current work, significantly larger modules operating in a cross-flow configuration were used.

Notably, with regard to the separation of fermentation broths, fouling may be a very complicated phenomenon due to the wide variety of foulants present in a feed. In the literature, fouling is generally described with the use of the following mechanisms: (i) cake formation, (ii) complete blocking, (iii) standard blocking, and (iv) intermediate blocking [15,16,17]. The cake layer is defined as a porous layer rejected on the membrane surface due to the adsorption, deposition, and accumulation of various foulants [18], which are composed of particles larger than the membrane pores’ size. In turn, with regard to complete blocking, it is assumed that feed particles block the membrane pores without overlapping with other particles. For the standard blocking, particles are smaller than membrane pores, and they are adsorbed and deposited within pores. As a consequence, the pore volume decreases automatically. Finally, for intermediate blocking, some particles deposit onto other ones while other particles block membrane pores. This occurs when the sizes of the particles and membrane pores are equivalent. During the filtration of broth containing significant amounts of microorganisms and suspended solids, in addition to membrane fouling, clogging of flow channels in modules can also occur. This issue is particularly relevant for spiral-wound modules [19,20]. It has been found that, with regard to capillary modules, this phenomenon may be prevented by using capillaries with diameters greater than 1.4 mm [21].

In addition to the clogging of module channels by sediments, a significant challenge is the decrease in membrane permeability caused by the fouling phenomenon. Accordingly, the implementation of strategies facilitating the minimization of fouling should be considered as a compulsory procedure. Among them, the most commonly used method is chemical cleaning, which utilizes specific agents, such as bases, acids, oxidizers, surfactants, chelating agents, and disinfectants, to restore the initial membrane performance [22]. Importantly, the used substances should be both effective and safe, as well as chemically stable and inexpensive [23]. Another important issue that must always be considered is that polymeric membranes may be subject to potential degradation during long exposure, leading to a reduction in their long-term performance and lifetime [24,25]. It can be caused by interaction between the membrane surface and both foulants and cleaning agents [26]. Consequently, the long-term use of MF membranes is still challenging since it requires the choice of the most suitable membrane material and the development of an effective membrane cleaning procedure to ensure stable system operation.

Alkaline cleaning agents are effective for cleaning membranes; however, due to their destructive properties, their concentration is limited to 0.5–1% NaOH [19,20,21]. On the other hand, fermentation solutions contain many components that create difficult-to-remove deposits on the membrane surface. Therefore, it is necessary to use solutions with a concentration of 1–3% NaOH, which may be used when chemically resistant membranes such as PP or PTFE are installed in the module [3,5,27,28].

Considering the above discussion, the main aim of the present work was to investigate the performance and stability of the long-term MF process of 1,3-PD fermentation broths using a capillary module. Additionally, channel blocking in spiral-wound modules was investigated. Fouling was mitigated by periodic washing with NaOH solutions. Due to their destructive effects, chemically resistant capillary PP and flat-sheet PTFE membranes were used in the work. It should be pointed out that these membranes are characterized by several notable advantages [29,30,31,32,33]; however, their hydrophobic properties may accelerate fouling, which was tested during their long-term use for the separation of fermentation broths.

## 2. Materials and Methods

### 2.1. Post-Fermentation Broths

In the current study, real 1,3-PD fermentation broths obtained via glycerol fermentation were used as a feed (Table 1). A strain of bacteria *Citrobacter freundii* was used for the production of 1,3-PD. To support their growth, in addition to salt, meat extract (1.5 g/L), yeast extract (2 g/L), and peptone K (2.5 g/L) were added to the broth, supplied by BTL (Poland). Their presence, along with bacteria, was the main cause of the membrane fouling phenomenon. The glycerol fermentation was thoroughly described in our several previous studies, e.g., [12,34]. After the fermentation process was complete, the stirrer in the bioreactor was switched off and the system was left for 2 h, allowing most of the suspensions to sediment. As a result, the turbidity of the fermentation broths was in the range from 1430 to 1700 NTU. The volume of the fermentation broths used as a feed for the MF process was equal to 3 L.

Studies on the glycerol fermentation showed that, in addition to 1,3-propanediol, carboxylic acids are also produced as byproducts. Their formation acidifies the broth, resulting in a decrease in fermentation efficiency. Hence, in the B1 and B3 fermentation broths, unfermented glycerol was present (Table 1). This can be avoided by stabilizing the broth pH at 7, which was achieved by adding a NaOH solution to the bioreactor. Notably, our previous work has shown that a NaOH solution previously used for cleaning membranes can be used for this purpose, eliminating waste generation in a membrane bioreactor [35].

### 2.2. Experimental Setup

**Figure 1:**
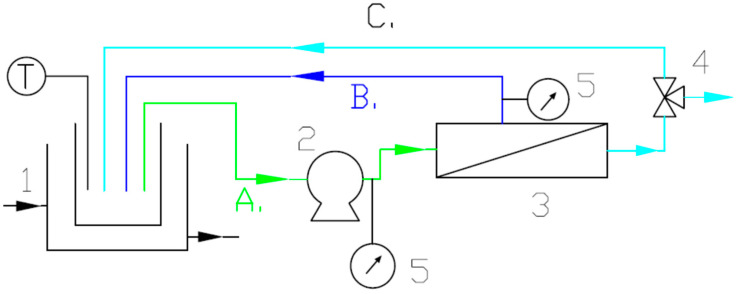
Experimental MF setup. 1—feed tank; 2—piston pump; 3—membrane module; 4—three-way valve; 5—manometer; T—electronic thermometer; A—feed; B—retentate; and C—permeate.

The MF process was carried out on the installation presented in Figure 1. Dedicated experimental investigations were performed at a temperature of 293 K, a feed flow rate equal to 0.38 L/min, and a transmembrane pressure (TMP) of 30 kPa.

For the MF studies, a capillary module and an additional flat module were used. The capillary module was made with the use of PP membranes Accurel PP S6/2 (Membrana GmbH, Wuppertal, Germany). They are hydrophobic (contact angle of 92°). The external and internal diameters of the membranes were equal to 2.6 mm and 1.8 mm, respectively. The membrane pores size was 0.2 µm. The module contained three capillaries with a length of 0.9 m (internal surface area of 152.7 cm^2^).

The MF experiments on flat-sheet membranes were carried out using a SEPA-CFII cross-flow module (GE Osmonics, Minnetonka, MN, USA). An active membrane area was 150 cm^2^, and the feed channel was filled by a polyethylene net (20 mesh). The construction of this module is recommended for testing the operation of spiral-wound modules. In the module for the MF tests, the microfiltration PTFE membrane Fluoropore FGLP (Millipore, Bedford, MA, USA) was mounted. The contact angle value for the new membrane used in this study was equal to 111 ± 2° which clearly indicates its hydrophobic nature. The membrane pore size was 0.22 µm. The total thickness of the membrane, including the supporting PP net, was to 200 µm.

Permeate flux *J* was determined based on the volume of permeate *V* passing through a unit membrane area *S* per unit time *t*, according to the following formula:

(1) $$J=\frac{V}{St}$$

### 2.3. Analytical Methods

The composition of fermentation broths was determined by high-performance liquid chromatography HPLC, using a UlitiMate 3000 (Thermo Fisher Scientific, Germering, Germany) and 850 Professional IC ion chromatograph with conductivity detector (Metrohm, Herisau, Switzerland), equipped with Metrohm A Supp5-250 and Metrosep C2-150 analytical columns. The feed and permeate turbidity were investigated with a portable turbidity meter model 2100 AN IS (Hach Company, Loveland, CO, USA). The MF membranes’ morphology and the composition of the deposit layer formed on their surface were investigated using a Hitachi SU70 and SU80 Scanning Electron Microscope (SEM) with energy-dispersive X-ray spectrometer (EDS) (Hitachi, Tokyo, Japan).

### 2.4. Membranes Cleaning

In the current study, in order to investigate the long-term performance of the PP membranes, MF of fermentation broths was conducted with repeated membrane cleaning. For this purpose, water and the 1% sodium hydroxide solution were used (Table 2).

### 2.5. Analysis of Fouling Mechanism

In order to determine dominant fouling mechanism during the MF of fermentation broths, the Hermia model was used [36]. The details of the analysis procedure have been demonstrated in our previous works [37,38]. The coefficient of determination R^2^ was used to assess the consistency of experimental data with numerical results (Table A1, Table A2, Table A3 and Table A4, presented in Appendix A).

## 3. Results and Discussion

### 3.1. Flat-Sheet Membrane

**Figure 2:**
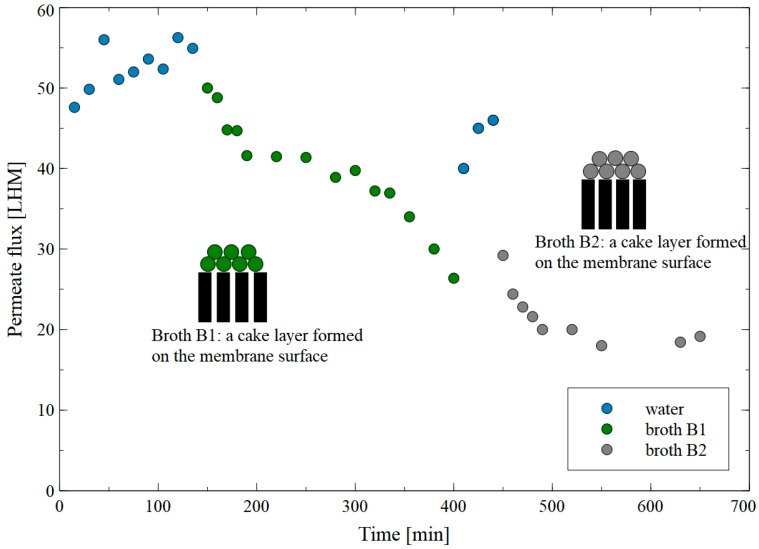
Changes in the permeate flux during MF of B1 and B2 fermentation broths with the use of the PTFE membrane.

It is well known that spiral-wound modules are used in many industrial membrane systems. However, as mentioned above, broth separation can cause rapid channel blocking in such modules. To determine whether the pretreatment of broth by sedimentation reduces this phenomenon, MF of B1 and B2 fermentation broths with the use of a flat-sheet PTFE membrane was conducted (Figure 2). The PTFE membrane is highly hydrophobic (a contact angle of 111°), which prevents water from flowing through air-filled pores. For this reason, the membrane was wetted with ethanol, followed by undergoing filtration with distilled water for 135 min under TMP of 0.15 MPa. This allowed the stable permeate flux equal to 55 LHM to be achieved. Subsequently, the MF of B1 fermentation broth for another 265 min was performed. It has been found that this led to a decrease in the flux from 50 LHM to 26.4 LHM. Undoubtedly, this finding clearly indicates the occurrence of the fouling phenomenon. Then, the membrane was washed by water rinsing for 40 min. It resulted in a slight increase in the membrane performance. Indeed, the noted flux was equal to 46 LHM indicating a significant contribution of irreversible fouling. The results obtained in the current study are in line with those demonstrated in [6], wherein it was documented that during the separation of 1,3-PD fermentation broths with the use of MF membranes made of PES and PP, the permeate flux decreased from about 500 LMH to 7.5 LMH and from about 410 LMH to 7 LMH, respectively.

In the second series of the MF run, the separation of the B2 broth with the recycling of a permeate to the feed tank was performed for 40 min. Notably, in this case, the permeate flux decreased from 29.2 LHM to a stable level of 20 LHM. This finding indicates that for the given process conditions (flow rate and feed turbidity), the cake thickness also stabilized. Notably, it is important to note that the analysis performed with the use of Hermia’s model allowed for the demonstration that, during MF of B1 and B2 fermentation broths, the formation of a cake layer on the membrane surface was a dominant mechanism of the fouling phenomenon (Table A1). This can be explained by the fact that *Citrobcater freundii* bacteria present in the feed are characterized by a length (1–5 μm) significantly bigger than the PTFE membrane pores’ size (0.2 µm). Moreover, it can be assumed that suspended colloids and particles present in the fermentation broths aggregated with each other and formed on the membrane surface as a cake layer. As it has been described by Shi et al. [39], the formation of a cake layer is a fouling mechanism in which particles accumulate layer by layer on the membrane surface, leading to additional resistance to the feed flow.

**Figure 3:**
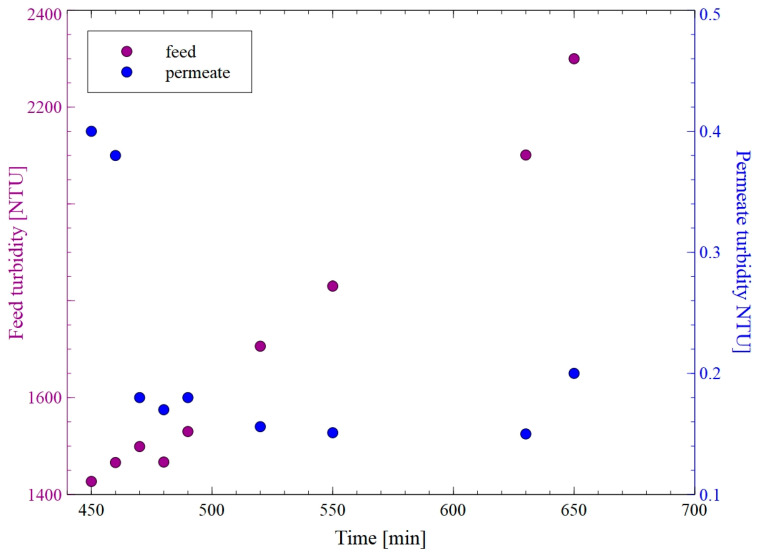
Changes in the feed and permeate turbidity during MF of B2 fermentation broth with the use of the PTFE membrane.

Figure 3 shows changes in the feed and permeate turbidity during MF of the B2 fermentation broth with the use of PTFEE membrane. The obtained results demonstrated that the initial permeate turbidity of 0.4 NTU decreased to 0.17 NTU. The decline in the permeate flux (Figure 2) and improved separation efficiency can be explained by the formation of a filter cake on the membrane surface. Indeed, as it has been indicated in [40], a deposited cake layer increases the hydraulic resistance to fluid flow, leading to a decrease in the permeate flux and an increase in the rejection of feed compounds, such as bacteria cells. Notably, permeability through the cake layer depends on several factors, including foulant size, deformability, and shape, as well as process conditions [41]. Later in the run, the broth was concentrated by doubling its volume. This resulted in an increase in feed turbidity from 1430 to 2300 NTU; nevertheless, the flux and separation degree remained almost constant. These findings clearly demonstrate that the PTFEE membrane can be successfully used for the long-term separation of fermentation broths, ensuring stable process performance and separation efficiency.

**Figure 4:**
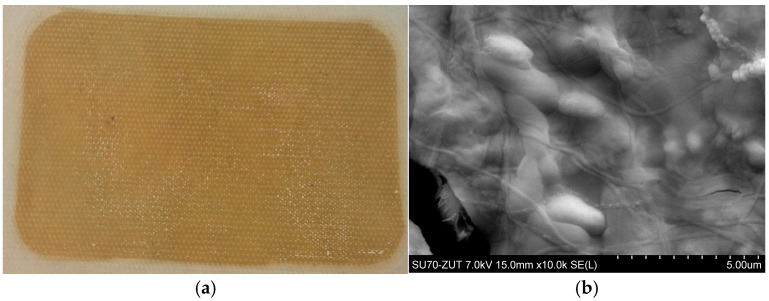
Image of the PTFE membrane surface after MF of B1 and B2 fermentation broths: (**a**) feed side, and (**b**) SEM image membrane surface with deposit.

After filtration, the PTFEE membrane surface was covered with a deposit, which confirmed the formation of a filter cake (Figure 4). Hence, it can be indicated that the use of flat membranes for broths’ separation in bioreactors may be a great challenge due to the significant fouling phenomenon. Notably, the formation of a cake layer during the MF of various types of fermentation broths has been reported for membranes made of ceramic [40], polyether sulfone (PES) [42], mixed cellulose ester [43], PP [6], and ceramic [14].

**Figure 5:**
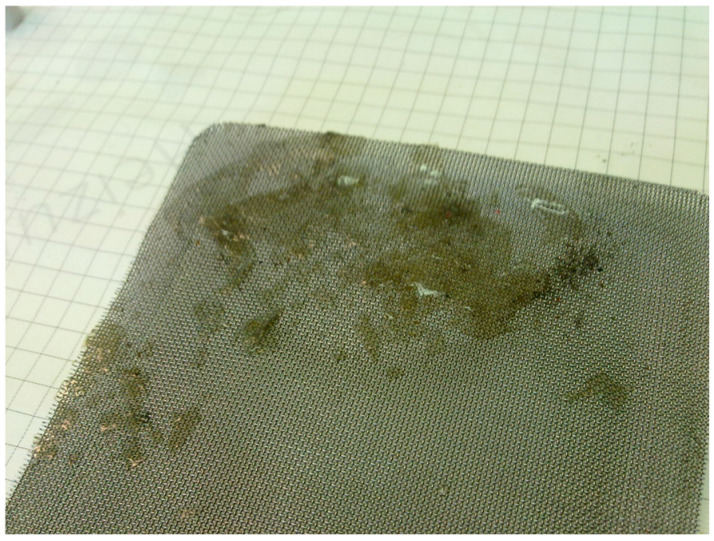
Image of the deposit formed inside the net filling the feed channel during filtration B1 and B2 broths.

A significant amount of deposit was also observed in the net filling the feed channel, particularly on the feed inlet side (Figure 5). It should be pointed out that the above-mentioned deposit was generated during the 700 min MF process. This finding indicates that in an industrial installation, channel blockage in spiral-wound modules can be expected after just a few days of the process run. Studies in an industrial installation have shown that, despite the use of complex membranes’ washing, it is difficult to clean such channels [20]. Therefore, fermentation broths’ pretreatment by sedimentation is insufficient, and an additional pre-filtration process must be implemented to enable the use of such modules used for MF broths.

### 3.2. Capillary Module

**Figure 6:**
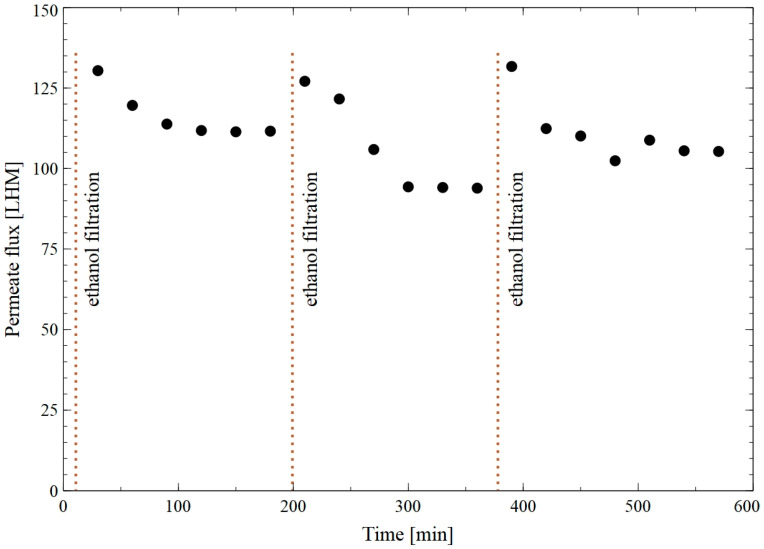
Changes in the water flux after wetting of PP membrane with the use of ethanol.

It is well known that capillary modules are recommended for the MF of fermentation broths [21]. Therefore, polypropylene capillary membranes formed using the TIPS method were also used in this study. As indicated above, the Accurel PP S6/2 capillary membranes are hydrophobic, hence, they were pre-wetted with ethanol. For this purpose, a filtration cycle was carried out consisting of successive and repeated stages, as follows: filtration of ethanol (10 min) and then distilled water (180 min) under TMP of 30 kPa. According to the data obtained, for the PP membranes, compared to the PTFE one, despite the lower TMP value, a significantly higher maximum flux was noted. Indeed, it was equal to 105 LHM (Figure 6).

**Figure 7:**
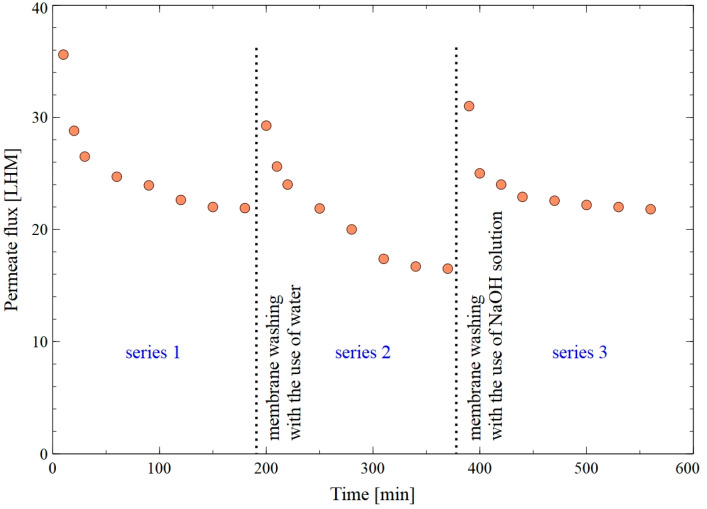
Changes in the permeate flux during MF of B3 fermentation broth with the use of the PP membrane.

The filtration of the B3 fermentation broth resulted in a significant decrease in the membrane performance (Figure 7). Analysis performed with the use of Hermia’s model showed that the dominant mechanism of the fouling phenomenon was the formation of a cake layer on the membrane surface (Table A2). It has been noted that the flux declined from 36 LMH to 22 LMH after 120 min of the process run (series 1). It should be pointed out that after rinsing the module with water, the flux increased to 30 LHM. However, continuing the MF of the broth led to the flux decline to 17 LHM (series 2). This result indicated that rinsing with water did not completely clean the membrane surface and did not ensure the high process performance. This can be explained by the fact that the fermentation broth consisted of multiple compounds not removable with water. It is evident from the available literature that the formation of a cake layer may be limited by the periodic chemical cleaning of the membrane. Hence, the membrane was cleaned with the use of 1% sodium hydroxide (NaOH) solution for 10 min. It has been found that this facilitated a significantly higher membrane performance (series 3). Indeed, at the end of the process run, the flux was equal to about 22 LMH. Roughly speaking, it can therefore be assumed that the chemical agent affected the bonds between the foulants and the membrane [44]. On other words, the NaOH solution could remove organic fouling via various mechanisms, such as promoting protein hydrolysis, electrostatic repulsion, membrane pore swelling, and foulants dissolution [45]. The results discussed above demonstrate that the applied method of membrane cleaning has a significant impact on the MF process performance. In addition, during the long-term MF of fermentation broths, membrane cleaning should be considered as an integral part of the system operation that must be conducted regularly.

**Figure 8:**
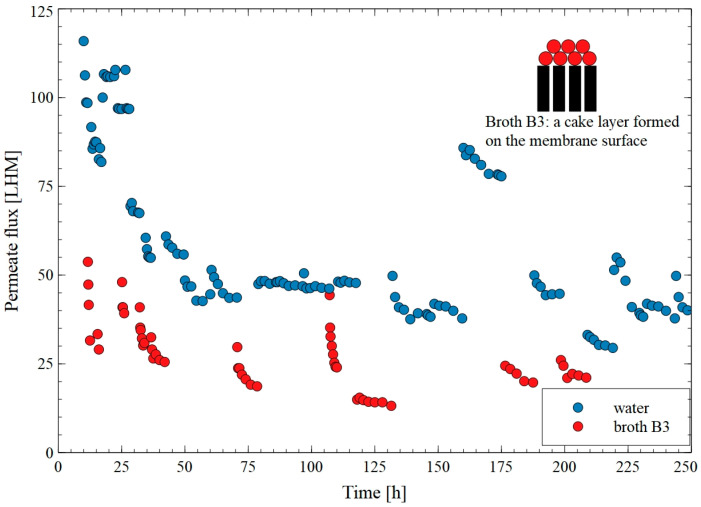
Changes in the permeate flux during long-term MF of B3 fermentation broth with the use of the PP membrane periodically washed with water.

The changes in the permeate flux during MF of the B4 fermentation broth with periodical membrane washing with water are shown in Figure 8. After the first two process cycles, washing the membranes with water effectively restored the initial performance. Indeed, the noted flux was equal to 110 LMH. However, from the 25th hour of the process run, after a subsequent series of broth separations, water washing became less effective, and the flux decreased to the value of 40 LMH. After 160 h, the flux increased to 80 LMH, which was due to the fact that the system was not operating, and the membranes were soaked in distilled water for seven days. This effect, however, was short-lived, and after two subsequent broth separation cycles, the water flux again decreased to 40 LMH. An analysis of the fouling mechanism indicated that during the first series (between 11.6 h and 21.5 h) of the long-term MF of B3 fermentation broth, the fouling was caused by various mechanisms, including complete, standard, and intermediate blocking, as well as a cake layer formation (Table A3). This finding clearly confirmed that, with regard to the MF of fermentation broths, fouling may be very complex phenomenon. In turn, in all subsequent stages of the process, a cake deposition was dominant. This finding confirmed the indication presented in [46], wherein it was pointed out that membrane fouling during the filtration of fermentation broth may be a combined phenomenon due to the fact that bacteria cell, proteins, broth medium, and fermentation products may have different fouling behaviors on the membrane surface.

**Figure 9:**
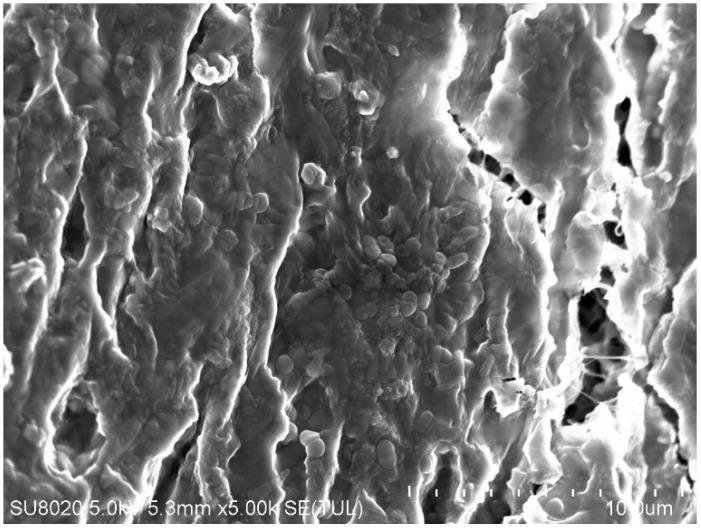
SEM image of the PP S6/2 membrane covered by deposit after MF of B3 fermentation broth (Figure 8).

SEM analysis of the PP membranes showed that after washing with water, almost the entire surface of the membranes was still covered with deposit (Figure 9).

As can be seen in Figure 9, after rinsing the membrane with water, numerous pores were visible in the deposit layer, allowing the feed to flow to the membrane surface and pores. Their size and number likely increased after the membrane rinsing, resulting in increased water flux (Figure 8). Moreover, the deposit layer was thin; thus, the blocking of the feed flow in the capillary (1.8 mm diameter) did not occur. Obviously, this is an advantage of capillary modules, in which there is no clogging of the nets filling the channels of the spirally wound module (Figure 5). However, regardless of the module type, during MF of broths, membrane fouling should be expected.

**Figure 10:**
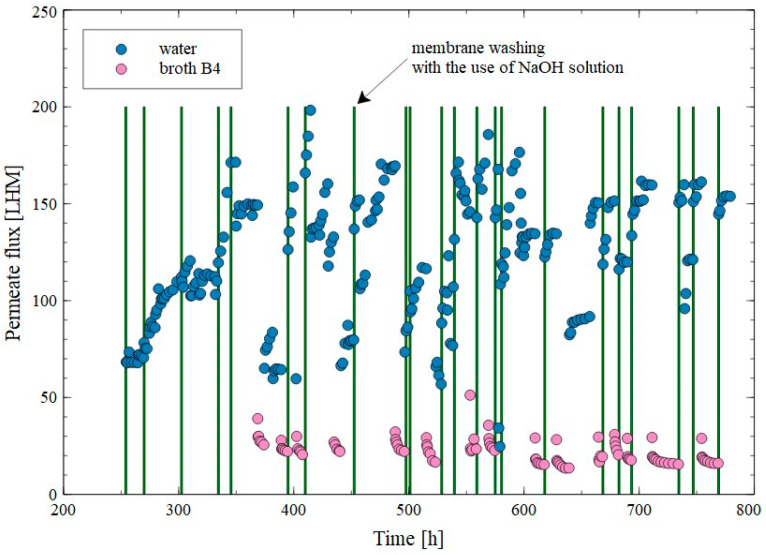
Changes in the permeate flux during long-term MF of B4 fermentation broth with the use of the PP membrane, periodically washed with the water and 1% NaOH solution.

To evaluate the performance of the PP membranes used for the separation of 1,3-PD fermentation broths, the long-term process was carried out for 780 h. Additionally, the membrane module was periodically washed with water and the 1% NaOH solution. During the first 100 h of the process run, membrane washing was performed to remove the foulants formed during previously performed MF process run (Figure 8). After the first three cycles of the membrane’s cleaning with 1% NaOH solution, the water flux increased to a value of 110 LMH corresponding to that reported for new membranes (Figure 10). Interestingly, after five cycles of washing, the water flux increased above 150 LMH, which could indicate an increase in the membrane’s porosity. As expected, during the MF of the fermentation broth, the flux decreased to 30 LMH. After rinsing the membrane with water, the flux was 64 LMH, which increased to 150–200 LMH in the following days after NaOH rinsing. Despite such intensive cleaning of the membrane, during broth separation, the performance in the range of 15–35 LMH was obtained. Importantly, these values were only slightly higher than those obtained when only membrane rinsing with water was used. Moreover, analysis performed with the use of Hermia’s model demonstrated that, during the long-term MF process, the formation of a cake layer was the dominant mechanism leading to a decrease in the permeate flux (Table A4).

**Figure 11:**
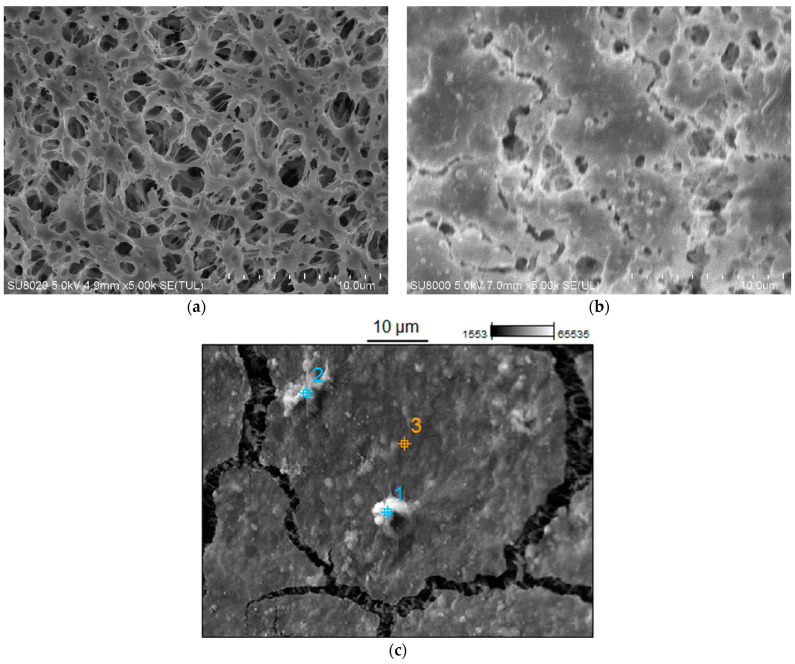
SEM images of the PP S6/2 membranes: (**a**) pristine membrane; (**b**) membrane with deposit formed during long-term MF of B4 fermentation broth with periodic membrane rinsing with 1% NaOH; and (**c**) locations of analyzed deposit shown in (**b**).

The morphology and structure of the pristine (Figure 11a) and fouled membrane (Figure 11b) were investigated by SEM analysis. The results obtained for membrane samples investigated after 778 h of the MF process run demonstrated that rinsing membranes with 1% NaOH solution did not remove all contaminants. Unfortunately, a residue remained on the membranes surface. The results of scanning electronic microscopy coupled with energy dispersive spectroscopy analyses demonstrated that the deposits located at the selected points (Figure 11c) consisted mainly of carbon, oxygen, and silicon, as well as smaller amounts of potassium and magnesium (Table 3).

As indicated above, during the initial period, an increase in permeate flux was observed after cleaning the membranes with the NaOH solution (Figure 10), which could have resulted from their alkaline degradation and increased pore size. In the subsequent period, despite repeated rinsing with the NaOH solution, this phenomenon was not observed. This could be due to the fact that the alkaline washing did not ensure completely removed deposits from the membrane surface (Figure 11), and its residues hindered the contact between PP and NaOH, which prevented polymer degradation.

**Figure 12:**
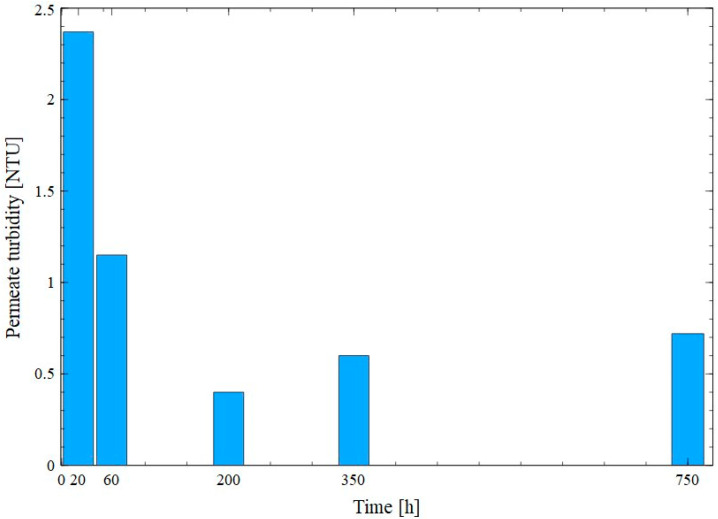
Changes in the permeate flux during long-term MF of B4 fermentation broth with the use of the PP membrane. Feed turbidity: 3500–3800 NTU.

Progressive membranes’ degradation may reduce separation efficiency. Hence, the quality of the permeate obtained during the MF process was systematically evaluated by determining its turbidity (Figure 12). It was observed during experimental investigation that initially, due to the formation of a filter cake, the turbidity of the permeate decreased from 2.37 NTU to 1.15 NTU. The lowest value of turbidity (0.4 NTU) was obtained at the end of the membrane cleaning period using only water (200 min of the process run), which confirms the significant impact of the cake layer, formed on the membrane surface, on the separation efficiency. Interestingly, washing the membranes with 1% NaOH solution removed some of the deposits, and as a result, the permeate turbidity increased to 0.6 NTU. Based on the results discussed above, it can be concluded that the membranes maintained their integrity and were resistant to frequent exposure to the foulants present in the fermentations broths and alkaline agent. To be complete, it should be noted that similar permeate turbidity values were obtained in other studies where the MF of fermentation broths was investigated [4,12].

**Figure 13:**
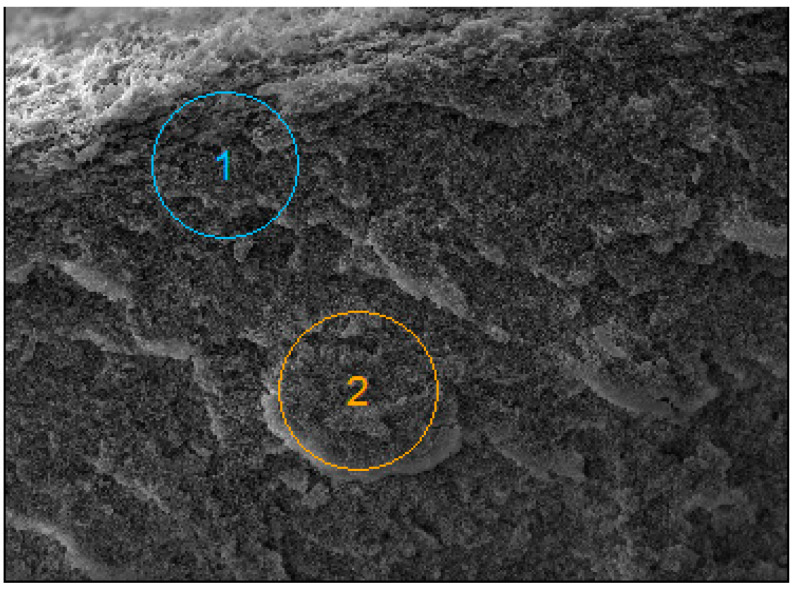
SEM images of the PP S6/2 membranes. Points 1 and 2: areas for the SEM-EDS analysis.

In addition to the formation of filter cake, deposits can also be present inside the membrane wall, which is the main cause of irreversible fouling. SEM-EDS analysis of a membrane cross-section sample performed after the completion of the MF process run (Figure 13) showed that only the pores closer to the membrane surface were blocked, and the quantity of contaminants decreased significantly 20–30 µm into the membrane wall (Table 4). This finding clearly confirmed that the formation of a cake layer was the dominant fouling mechanism (Table A3).

## 4. Conclusions

The current study offers a key critical insight into the application of capillary and spiral-wound modules with polymeric membranes for the long-term MF of fermentation broths. The MF modules were fed by broth pretreated only by 2 h sedimentation. The obtained results demonstrated that the applied capillary PP and flat-sheet PTFE membranes were efficient, achieving almost 100% recovery of suspended solids from the broth. Consequently, the obtained high-quality permeate was characterized by the turbidity of 0.4–0.7 NTU. However, the fouling phenomenon was the main limitation leading to the significant decrease in the process performance. Through detailed studies, we showed that the flux decline was mainly caused by the formation of a cake layer on the membranes’ surface. Experimental investigations with the use of a flat module showed that large amounts of deposits also formed in the net-filled channel. This result indicated that broth pretreated by sedimentation is insufficient to feed the spiral-wound modules. On the other hand, such issues were not noted upon the use of a capillary PP module. For the purpose of the mitigation of PP membrane fouling during the long-term MF, periodic cleaning was investigated. It has been demonstrated that rinsing the membranes with water did not remove the deposit layer formed on the membranes. Importantly, most of the foulants were removed by a cleaning system with the 1% NaOH solution. However, in this case, residual deposits formed by silicates remained on the membranes’ surface, which probably limited the membrane degradation during its cleaning with a NaOH solution. Finally, it is reasonable to conclude that the PP membranes used in the present study showed an excellent resistance to the frequent exposure to the foulants present in the fermentation broths and the alkaline agent.

## Figures and Tables

**Table 1:** The composition of post-fermentation broths.

Component [g/L]	B1	B3	B2	B4
1,3-propanediol	3.51	7.97	2.65	10.98
glycerol	5.23	2.87	5.56	0.25
lactic acid	0.69	2.48	0.77	1.67
acetic acid	0.81	1.52	0.92	2.63
succinic acid	0.31	0.78	0.33	1.43
Cl^−^	0.04	0.10	0.04	0.04
PO_4_^3−^	2.31	2.39	2.51	2.04
SO_4_^2−^	1.42	1.75	1.54	1.53
NH_4_^+^	0.57	0.58	0.59	0.56
K^+^	1.67	2.10	1.83	1.36
Na^+^	0.39	2.10	0.56	2.63
Ca^2+^	0.02	0.05	0.03	0.03
Mg^2+^	0.05	0.04	0.04	0.03
pH	5.55	6.69	5.21	7.02

**Table 2:** Procedure of the PP membranes’ cleaning.

Step	Cleaning Agent	Additional Information
1	water	After MF of the broth (3–4 h), the membrane module was flushed with water (3 L passed through the system). Subsequently, the module was supplied with distilled water, and the water flux was determined.
2	1% NaOH solution	A total of 2 L of the 1% NaOH solution was recirculated for 10 min, after which it was flushed with 3 L of water. Then, the system was fed with distilled water, and MF was carried out to determine the water flux.

**Table 3:** SEM-EDS analysis of the composition of a deposit layer formed on the PP membranes surface after long-term MF of B4 fermentation broth with periodic membrane rinsing with 1% NaOH [wt%].

Point	C	N	O	Mg	Si	K	Fe
1	47.80	-	43.20	0.51	4.45	2.46	1.28
2	36.59	7.87	44.50	0.18	9.88	0.98	-
3	68.13	-	27.23	0.52	4.12	-	-

**Table 4:** SEM-EDS analysis of the areas shown in Figure 13.

Point	C	O	Na	Mg	Si	Cl	Na
1	83.72	8.35	2.09	0.10	0.35	3.20	2.18
2	99.10	-	0.38	0.09	0.09	0.08	0.26

## Data Availability

The original contributions presented in the study are included in the article; further inquiries can be directed to the corresponding author.

## Appendix A

**Table A1:** Coefficient of determination (R^2^) Hermia’s model. MF of B1 and B2 fermentation broths with the use of the PTFE membrane (Figure 2).

Fermentation Broth	Complete Blocking	Standard Blocking	Intermediate Blocking	Cake Formation
B1 broth	0.8767	0.8553	0.8292	0.8667 *
B2 broth	0.5415	0.5589	0.5746	0.6004 *

* Indicates the best fitting model.

**Table A2:** Coefficient of determination (R^2^) Hermia’s model. MF of B3 fermentation broth with the use of the PP membrane (Figure 6).

MF Period	MF Time [min]	Complete Blocking	Standard Blocking	Intermediate Blocking	Cake Formation
series 1	0–180	0.7361	0.7666	0.7955	0.8467 *
series 2	200–370	0.9286	0.9432	0.9546	0.9687 *
series 3	390–560	0.5808	0.6025	0.6243	0.6675 *

* Indicates the best fitting model.

**Table A3:** Coefficient of determination (R^2^) Hermia’s model. MF of B3 fermentation broth with the use of the PP membrane periodically washed with water. (Figure 7).

MF Period	MF Time [h]	Complete Blocking	Standard Blocking	Intermediate Blocking	Cake Formation
series 1	11.6–12.5	0.9921 *	0.9971 *	0.9992 *	0.9957 *
series 2	25.2–26.0	0.6011	0.6106	0.6203	0.6402 *
series 3	32.1–34.0	0.7333	0.7481	0.7619	0.7869 *
series 4	36.7–42.0	0.5841	0.5958	0.6074	0.6320 *
series 5	70.7–78.5	0.7454	0.7767	0.8061	0.8581 *
series 6	107.0–110.0	0.8376	0.8672	0.8967	0.9387 *
series 7	118.0–131.5	0.8600	0.8615	0.8627	0.8637 *
series 8	176.5–187.5	0.8535	0.8542	0.8546	0.8548 *
series 9	198.5–208.5	0.5573	0.5571	0.5566	0.7548 *

* Indicates the best fitting model.

**Table A4:** Coefficient of determination (R^2^) Hermia’s model. MF of B4 fermentation broth with the use of the PP membrane periodically washed with the 1% NaOH solution and water (Figure 9).

MF Period	MF Time [h]	Complete Blocking	Standard Blocking	Intermediate Blocking	Cake Formation
series 1	368.7–374.0	0.5788	0.6074	0.6363	0.6937 *
series 2	389.1–394.5	0.4783	0.4917	0.5053	0.5356 *
series 3	402.5–407.5	0.7869	0.8062	0.8252	0.8612 *
series 4	435.0–440.0	0.9360	0.9399	0.9435	0.9501 *
series 5	488.1–496.0	0.7707	0.7904	0.8091	0.8432 *
series 6	515.1–523.0	0.9061	0.9239	0.9381	0.9568 *
series 7	553.1–558.5	0.8399	0.8274	0.8745	0.8889 *
series 8	569.1–574.5	0.6717	0.7013	0.7307	0.7863 *
series 9	609.7–617.5	0.6104	0.6430	0.6473	0.6480 *
series 10	628.2–639.0	0.4910	0.5416	0.5928	0.6905 *
series 11	664.7–668.0	0.8987	0.8829	0.8677	0.8905 *
series 12	678.7–682.0	0.8989	0.9178	0.9348	0.9626 *
series 13	689.7–693.0	0.6209	0.6374	0.6550	0.6928 *
series 14	711.2–734.0	0.6394	0.6839	0.7295	0.7400 *
series 15	754.2–768.5	0.6181	0.6551	0.6936	0.7719 *

* Indicates the best fitting model.
